# Four common vitamin D receptor polymorphisms and coronary artery disease susceptibility: A trial sequential analysis

**DOI:** 10.1371/journal.pone.0275368

**Published:** 2022-10-03

**Authors:** Xiaofei Yan, Yuzhen Wei, Dan Wang, Jiangtao Zhao, Kui Zhu, Yuan Liu, Hailong Tao

**Affiliations:** Department of Cardiology, The First Affiliated Hospital of Zhengzhou University, Zhengzhou, Henan, China; King Saud University, SAUDI ARABIA

## Abstract

**Background:**

Studies on the susceptibility of vitamin D receptor (VDR) polymorphisms to coronary artery disease (CAD) reached controversial results. We performed this study for a more accurate evaluation between the VDR polymorphisms and CAD susceptibility.

**Methods:**

PubMed, Embase, CNKI, Wan Fang, and VIP databases were searched. The odds ratios (ORs) and 95% confidence intervals (95% CIs) were used to evaluate the associations. Trial sequential analysis (TSA) was introduced to estimate the positive associations. The potential functions of the VDR polymorphisms were analyzed based on the SNPinfo and ENSEMBL databases.

**Results:**

Thirteen studies were finally included. In the overall analysis, increased CAD risks were observed in the VDR rs1544410 polymorphism and verified by the TSA; for the rs2228570 and rs731236 polymorphisms, significant associations with high heterogeneity were detected; decreased risk was remarkably observed for the rs7975232 polymorphism. In the subgroup analysis, wide associations with reduced heterogeneity were observed in the rs2228570, rs1544410, and rs731236 polymorphisms. The RNAfold analysis indicated the mutant G allele of the rs1544410 polymorphism was easier to disperse from the DNA double helix structure and may have a potential crucial role in the VDR transcription process.

**Conclusions:**

Our analysis supports the role of the rs1544410 polymorphism in the VDR gene as a risk factor for CAD. The VDR rs2228570 and rs731236 polymorphisms were associated with increased CAD risks in the White population. Restrict decreased CAD risk was firstly discovered in the rs7975232 polymorphism.

**Limitations:**

Firstly, the language was restricted to English and Chinese, which will cause the limited number of studies included; secondly, other unknown polymorphisms in VDR polymorphisms could also be associated the CAD susceptibility, and more case-control studies with comprehensive clinical outcomes and GWAS studies were required; thirdly, the rs1544410, rs7975232 and rs731236 polymorphism are in strong LD, haploid factors with CAD risk need to be considered; fourthly, the mechanisms of the VDR polymorphism on the VDR gene or RNA or protein were not discussed enough, further mechanistic studies are required; at last, genetic factor was the one side for CAD susceptibility, the interaction between environmental risk factors should be considered.

## Introduction

Risk factors have always been a hot topic in the study of coronary artery disease (CAD), which is the main leading cause of death in the world [1–3]. Traditional risk factors, such as smoking, obesity, high blood lipids, etc. [4], help physicians guide the population to prevent CAD. Whether genetic factors have an influence on the CAD risk remains unclear. A recent study reported the heritability of CAD has been estimated between 40% and 60% [5], which implied that genetic factors would play a distinctive role in the susceptibility of CAD.

Reduced serum vitamin D concentration was reported to be an increased risk marker for CAD [6], Vitamin D receptor (VDR) is a vital signal transduction molecule for vitamin D [7]. The *VDR* gene is located on chromosome 12q13.1, and has four common single nucleotide polymorphisms (SNPs) which are rs2228570 (FokI F/f in exon 2), rs1544410 (BsmI B/b in intron 8), rs7975232 (ApaI A/a in intron 8) and rs731236 (TaqI T/t in exon 9) [8]. Van Schooten et al. firstly reported the rs1544410 polymorphism was associated with the severity of CAD [9]. A small group study then conducted by Ortlepp et al. also confirmed the former results in 2001 [10], but in a larger population study reported by him in 2003, no association was detected [11]. In the next decades, many studies were designed and conducted not only in the rs1544410 polymorphism but also in other *VDR* polymorphisms, however the conclusions were inconsistent.

We considered the inconsistence may owe to the bias in sample size, different characteristics of research population or the unavoidable system errors, therefore, a comprehensive study based on rigorous inclusion and exclusion criteria was performed, and trial sequential analysis was introduced to reduce the system errors and confirm our positive results, moreover, the function of the *VDR* polymorphisms were analyzed with bioinformatic tools.

## Materials and methods

Based on the PRISMA checklist, we constructed the study [12].

### Identification of the related studies

Foreign (Embase, PubMed) and Chinese (China National Knowledge Infrastructure, VIP, and Wan fang) databases were thoroughly searched before Feb 28^th^ 2022. The terms “coronary artery disease,” “coronary heart disease,” “cardiovascular disease” “vitamin D receptor,” “VDR,” “variant,” “polymorphism,” and “polymorphisms” were used for constructing our searching strategy. Each author independently reviewed the potential studies and the divergence were discussed by group-meeting held by Hailong Tao (The corresponding author).

### Inclusion and exclusion criteria

Studies included in our study must meet the following inclusion criteria: (1) evaluation of the associations between the *VDR* polymorphisms and coronary artery disease susceptibility; (2) case-control study or cohort design; (3) detailed genotype frequency data could be acquired to calculate odds ratios (ORs), 95% confidence intervals (CIs) and Hardy Weinberg Equilibrium test; Exclusion criteria: (1) duplication of previous publications; (2) comment, review, case reports, animal studies and editorials; (3) study with no detailed genotype frequency data. The first two authors conducted the selection of potential included studies independently based on the inclusion and exclusion criteria. Any disagreement was solved by a discussion with the corresponding author.

### Data extraction

For each study, the following data were independently extracted by the first two authors and the corresponding author used a standardized form: first author’s last name, year of publication, study country, region, age, BMI, 25(OH) vitamin D, genotyping methods, detail genotype frequency data of cases and controls, genotype distribution in CAD (coronary artery disease) populations and controls, quality score and the result of Hardy-Weinberg Equilibrium test.

### Quality score assessment

The modified Newcastle-Ottawa scale (NOS) was used to evaluate the quality of included studies in our study (S2 Table) [13–16]. Each included study was scored and regarded as either low quality (score ≤ 6) or high quality (score > 6) based on items such as the definition of representativeness of cases, source of controls, sample size, quality control of genotyping method, and Hardy-Weinberg equilibrium.

### Statistics analysis

Review Manager, Version 5.3 (The Nordic Cochrane Centre, The Cochrane Collaboration; Copenhagen, Denmark) and STATA 12.0 (STATA Corp, LP) were used for all analysis. P < 0.05 was considered to be significant. Hardy–Weinberg equilibrium (HWE) was evaluated for each study by Chi-square test in control groups, and P < 0.05 was considered as a significant departure from HWE. Odds ratio (OR) and 95% confidence intervals (CIs) were calculated. The odds ratio (OR) is the ratio of odds of an event in one group versus the odds of the event in the other group. An odds ratio (OR) of 1.0 indicates that there is no difference in risk (or odds) between the groups being compared. An OR of more than 1.0 indicates an increase in risk (or odds) among the exposed compared to the unexposed, whereas an OR <1.0 indicates a decrease in risk (or odds) in the exposed group [17]. Pooled ORs were performed in allelic model (rs2228570 polymorphism: F versus f; rs1544410 polymorphism: B versus b; rs7975232 polymorphism: A versus a; rs731236 polymorphism: T versus t), recessive model (rs2228570 polymorphism: FF versus Ff+ff; rs1544410 polymorphism: BB versus Bb+bb; rs7975232 polymorphism: AA versus Aa+aa; rs731236 polymorphism: TT versus Tt+tt); dominant model (rs2228570 polymorphism: FF+Ff versus ff; rs1544410 polymorphism: BB+Bb versus bb; rs7975232 polymorphism: AA+Aa versus aa; rs731236 polymorphism: TT+Tt versus tt); heterozygote model (rs2228570 polymorphism: Ff versus ff; rs1544410 polymorphism: Bb versus bb; rs7975232 polymorphism: Aa versus aa; rs731236 polymorphism: Tt versus tt); homozygote model (rs2228570 polymorphism: FF versus ff; rs1544410 polymorphism: BB versus bb; rs7975232 polymorphism: AA versus aa; rs731236 polymorphism: TT versus tt), respectively.

Heterogeneity was evaluated by Q statistic (significance level of P < 0.1) and I^2^ statistic (greater than 50% as evidence of significant inconsistency). If the P value for heterogeneity was >0.10 and I2 <50%, indicating an absence of heterogeneity between studies, the fixed-effects model (the Mantel-Hasenszel method) would be used; If the P value for heterogeneity was ≤0.10 or I2 ≥50%, indicating a high exist of heterogeneity between studies, and the random-effects model (the DerSimonian and Laird method) would be used. Besides, subgroup analyses were stratified by Race (White, Asian and African), Hardy-Weinberg equilibrium (in accordance with HWE, departure from HWE), sample size (≥500, <500), genotyping method (PCR-RFLP, PCR-Taqman, PCR-ABD), and random-effects model were applied in subgroup analysis for more conservative results. We applied the Bonferroni method, which controls for the false discovery rate (FDR), to adjust for multiple comparisons.

### Trial sequential analysis (TSA)

TSA (The Copenhagen Trial Unit, Center for Clinical Intervention Research, Denmark) is a methodology that combines an information size calculation (cumulated sample sizes of all included trials) for a meta-analysis with the threshold of statistical significance (http://www.ctu.dk/tsa). If the data in included studies are sparse or if there is repeated testing for significance in conducting an updated meta-analysis, the type I errors and type II errors are unavoidable [18, 19].

To reduce the risk of type I errors, TSA was introduced in our analysis. The required information size was calculated according to an overall type-I error of 5%, a power of 80% and a relative risk reduction (RRR) assumption of 20% [20, 21]. A continuity correction of 0.5 was also applied in zero-event trials [22].

### Bioinformatics analysis

Ensembl is a genome browser for vertebrate genomes that supports research in comparative genomics, evolution, sequence variation and transcriptional regulation, and this database provides the genomic context, genes and regulatory elements, flanking sequence, population genetics, phenotype data, sample genotypes, linkage disequilibrium and phylogenetic context of a single nucleotide polymorphism (http://asia.ensembl.org/index.html). SNPinfo is an important bioinformatics analysis tool that predicts SNP function. The SNPinfo database can help researchers specify genes or linkage regions and select SNPs based on GWAS results, calculate linkage disequilibrium (LD), and predict functional characteristics of both coding and non-coding SNPs (https://snpinfo.niehs.nih.gov/) [23]. In addition, the RNAfold web server is one of the core programmes of the Vienna RNA package that has been used to predict the minimum free energy of single sequences that influence the stability of the structure [24]. Therefore, we conducted bioinformatics analyses using the above databases and methods to identify the potential molecular mechanisms for further research.

## Results

The PRISMA flow diagram of the literature selection process was showed in S1 Table.

### The characteristics of included studies

Thirteen studies were finally included in our manuscript [11, 25–36]. Table 1 summarized the characteristics of the included studies. For the rs2228570 polymorphism, a total of eleven studies were included in the study with 1908 CAD patients and 1923 controls [25–27, 29–32, 34, 36]; ten studies were analyzed for the study including 4210 CAD patients and 10004 controls for the rs1544410 polymorphism [11, 25, 27–29, 35, 36]; for the rs731236 polymorphism, 3136 CAD patients and 9501 controls were included [25–27, 29, 30, 32, 33, 35]; for the rs7975232 polymorphism, nine studies were included with 2815 CAD patients and 9460 controls [26, 27, 29, 32, 33, 35].

**Table 1:** Characteristics of included studies about the four VDR polymorphisms and coronary artery disease.

				Age(year)		BMI(Kg/m2)		25(OH) vitamin D(ng/ml)		Genotyping	CAD				Control				Quality	
**Author**	**Year**	**Country**	**Region**	**CAD**	**Control**	**CAD**	**Control**	**CAD**	**Control**	**Method**	**11**	**12**	**22**	**N**	**11**	**12**	**22**	**N**	**Score**	HWE*
**rs2228570 polymorphism**																				
Raljevic	2021	Croatia	Rijeka	58 ± 11	47 ± 11	28.5 ± 4.0	27.2 ± 4.5	NA	NA	PCR-RFLP	50	84	21	155	34	52	18	104	9	0.805
Fronczek	2021	Poland	Zabrze	NA	NA	25.3 ± 4.6	23.4 ± 4.1	23:50 ± 11:08	21.76 ± 10.37	PCR-Taqman	105	186	95	386	139	242	78	459	9	0.116
Ma	2020	China	Xian	60.3 ± 2.3	62.4 ± 3.3	NA	NA	NA	NA	PCR-RFLP	44	88	6	138	344	161	16	521	9	0.586
Moradi	2017	Iran	Tehran	59.37 ± 1.04	56.16 ± 1.28	26.61 ± 0.35	26.89 ± 0.44	10.2 (8.2–19.1)	37.5 (14.1–52.6)	PCR-RFLP	44	15	45	104	42	4	23	69	7	0.000
Maia	2016	Brazil	Pernambuco	65.7 (±7.18)	65.1 (±9.18)	NA	NA	NA	NA	PCR-Taqman	12	4	2	18	34	38	10	82	8	0.902
Maaty	2016	Egypt	Cairo	NA	NA	NA	NA	NA	NA	PCR-Taqman	52	31	15	98	24	13	18	55	7	0.000
He	2015	China	Nanchang	62.14 ± 9.40	59.64 ± 13.31	NA	NA	NA	NA	PCR-ABD	61	103	51	215	28	27	12	67	9	0.234
Hossein-Nezhad 1	2014	Iran	Tehran	55.96 ± 12.48	57.46 ± 10	NA	NA	8.03 ± 5.35	8.17 ± 5.06	PCR-RFLP	104	82	16	202	76	34	8	118	9	0.136
Hossein-Nezhad 2	2014	Iran	Tehran	58.42 ± 9.80	57.46 ± 10	NA	NA	7.68 ± 5.83	8.17 ± 5.06	PCR-RFLP	82	74	12	168	76	34	8	118	8	0.136
Hossein-Nezhad 3	2014	Iran	Tehran	59.41 ± 9.49	57.46 ± 10	NA	NA	5.77 ± 5.24	8.17 ± 5.06	PCR-RFLP	152	98	22	272	76	34	8	118	9	0.136
Pan	2009	China	Chengdu	NA	NA	NA	NA	NA	NA	PCR-RFLP	47	65	40	152	68	97	47	212	9	0.270
**rs1544410 polymorphism**																				
Raljevic	2021	Croatia	Rijeka	58 ± 11	47 ± 11	28.5 ± 4.0	27.2 ± 4.5	NA	NA	PCR-RFLP	72	60	23	155	39	59	6	104	8	0.008
Ma	2020	China	Xian	60.3 ± 2.3	62.4 ± 3.3	NA	NA	NA	NA	PCR-RFLP	115	19	4	138	443	63	15	521	9	0.000
Kiani	2019	Iran	Kermanshah	63.4 ± 8.8	64.09 ± 7.5	24.9 ± 2.9	24.4 ± 2.5	13.5 (7.75–21.2)	16.2 (5.2–26.2)	PCR-RFLP	40	69	48	157	69	79	34	182	8	0.185
Moradi	2017	Iran	Tehran	59.37 ± 1.04	56.16 ± 1.28	26.61 ± 0.35	26.89 ± 0.44	10.2 (8.2–19.1)	37.5 (14.1–52.6)	PCR-RFLP	43	17	44	104	38	3	28	69	7	0.000
Ferrarezi 1	2013	Brazil	Paulo	68 ± 8	65 ± 8	29.2 ± 4.4	29.4 ± 4.6	NA	NA	PCR-ABD	154	242	86	483	969	1261	425	2654	9	0.663
Ferrarezi 2	2013	Brazil	Paulo	68 ± 8	65 ± 8	29.2 ± 4.5	29.4 ± 4.7	NA	NA	PCR-ABD	138	246	81	465	986	1256	430	2672	9	0.370
Ferrarezi 3	2013	Brazil	Paulo	68 ± 8	65 ± 8	29.2 ± 4.6	29.4 ± 4.8	NA	NA	PCR-ABD	248	418	139	805	875	1084	373	2332	9	0.225
Ferrarezi 4	2013	Brazil	Paulo	67 ± 10	61 ± 11	29.0 ± 5.2	28.5 ± 5.5	NA	NA	PCR-ABD	64	119	47	230	151	251	81	483	9	0.175
Pan	2009	China	Chengdu	NA	NA	NA	NA	NA	NA	PCR-RFLP	2	21	129	152	4	38	170	212	8	0.285
Ortlepp	2003	Germany	Aachen	68.0 ± 6.7	56.5 ± 11.2	NA	NA	NA	NA	PCR-RFLP	249	794	481	1524	124	419	232	775	7	0.004
**rs7975232 polymorphism**																				
Fronczek	2021	Poland	Zabrze	NA	NA	25.3 ± 4.6	23.4 ± 4.1	23:50 ± 11:08	21.76 ± 10.37	PCR-Taqman	97	196	93	386	114	213	129	456	9	0.167
Ma	2020	China	Xian	60.3 ± 2.3	62.4 ± 3.3	NA	NA	NA	NA	PCR-RFLP	58	63	17	138	203	224	94	521	9	0.021
Moradi	2017	Iran	Tehran	59.37 ± 1.04	56.16 ± 1.28	26.61 ± 0.35	26.89 ± 0.44	10.2 (8.2–19.1)	37.5 (14.1–52.6)	PCR-RFLP	57	10	37	104	38	9	22	69	7	0.000
He	2015	China	Nanchang	62.14 ± 9.40	59.64 ± 13.31	NA	NA	NA	NA	PCR-ABD	7	29	31	67	18	93	104	215	8	0.801
Maaty	2015	Egypt	Cairo	NA	NA	NA	NA	9.99±1.32	35.92±3.9	PCR-Taqman	38	63	36	137	22	22	14	58	8	0.637
Ferrarezi 1	2013	Brazil	Paulo	68 ± 8	65 ± 8	29.2 ± 4.4	29.4 ± 4.6	NA	NA	PCR-ABD	137	235	112	483	679	1369	605	2654	9	0.091
Ferrarezi 2	2013	Brazil	Paulo	68 ± 8	65 ± 8	29.2 ± 4.4	29.4 ± 4.6	NA	NA	PCR-ABD	120	248	97	465	697	1355	620	2672	9	0.436
Ferrarezi 3	2013	Brazil	Paulo	68 ± 8	65 ± 8	29.2 ± 4.4	29.4 ± 4.6	NA	NA	PCR-ABD	215	414	176	805	602	1189	541	2332	9	0.324
Ferrarezi 4	2013	Brazil	Paulo	67 ± 10	61 ± 11	29.0 ± 5.2	28.5 ± 5.5	NA	NA	PCR-ABD	86	101	42	230	135	229	118	483	9	0.324
**rs731236 polymorphism**																				
Raljevic	2021	Croatia	Rijeka	58 ± 11	47 ± 11	28.5 ± 4.0	27.2 ± 4.5	NA	NA	PCR-RFLP	76	57	22	155	45	54	5	104	8	0.026
Fronczek	2021	Poland	Zabrze	NA	NA	25.3 ± 4.6	23.4 ± 4.1	23:50 ± 11:08	21.76 ± 10.37	PCR-Taqman	152	180	54	386	188	209	62	459	9	0.746
Ma	2020	China	Xian	60.3 ± 2.3	62.4 ± 3.3	NA	NA	NA	NA	PCR-RFLP	39	94	5	138	333	172	16	521	9	0.269
Moradi	2017	Iran	Tehran	59.37 ± 1.04	56.16 ± 1.28	26.61 ± 0.35	26.89 ± 0.44	10.2 (8.2–19.1)	37.5 (14.1–52.6)	PCR-RFLP	52	18	34	104	33	10	26	69	7	0.000
Maia	2016	Brazil	Pernambuco	65.7 (±7.18)	65.1 (±9.18)	NA	NA	NA	NA	PCR-Taqman	8	7	3	18	37	39	6	82	8	0.320
He	2015	China	Nanchang	62.14 ± 9.40	59.64 ± 13.31	NA	NA	NA	NA	PCR-ABD	195	20	0	215	63	4	0	67	9	0.955
Maaty	2015	Egypt	Cairo	NA	NA	NA	NA	9.99±1.32	35.92±3.9	PCR-Taqman	36	60	41	137	18	27	13	58	8	0.637
Ferrarezi 1	2013	Brazil	Paulo	68 ± 8	65 ± 8	29.2 ± 4.4	29.4 ± 4.6	NA	NA	PCR-ABD	163	240	81	483	982	1266	406	2654	9	0.951
Ferrarezi 2	2013	Brazil	Paulo	68 ± 8	65 ± 8	29.2 ± 4.4	29.4 ± 4.6	NA	NA	PCR-ABD	153	247	66	465	994	1256	422	2672	9	0.446
Ferrarezi 3	2013	Brazil	Paulo	68 ± 8	65 ± 8	29.2 ± 4.4	29.4 ± 4.6	NA	NA	PCR-ABD	265	415	126	805	881	1089	361	2332	9	0.420
Ferrarezi 4	2013	Brazil	Paulo	67 ± 10	61 ± 11	29.0 ± 5.2	28.5 ± 5.5	NA	NA	PCR-ABD	80	110	40	230	199	212	73	483	9	0.420

For FokI polymorphism, 11, 12 and 22 represent FF, Ff, ff, respectively; for BsmI polymorphism, 11, 12 and 22 represent BB, Bb and bb, respectively; for ApaI polymorphism, 11, 12 and 22 represent AA, Aa and aa, respectively

for TaqI polymorphism, 11, 12 and 22 represent TT, Tt, tt, respectively.

For the study of Hossein-Nezhad et al., the author divided the CAD populations into three groups based on the numbers of arteries in luminal stenosis, so there are three small sub-studies from Hossein-Nezhad et al.

For the study of Ferrarezi et al., two cohort studies (DIABHYCAR and NCH) were included in the study, and the former cohort study had three independent sub-studies on this topic, so there are four sub-studies from Ferrarezi et al.

* P value for Hardy–Weinberg equilibrium test in controls; NA = Not available; CAD = Coronary Artery Disease; VDR = Vitamin D receptor; PCR-RFLP = polymerase chain reaction-restriction fragment length polymorphism.

PCR-Taqman = polymerase chain reaction with Taqman probe; PCR-ABD = polymerase chain reaction using Assay by Design (ABD) kits from Applied Biosystems (Carlsbad, CA, USA).

### The analysis of VDR polymorphisms and CAD susceptibility

The results of overall and subgroup populations were showed in Table 2.

**Table 2:** Overall and subgroup analysis of the associations of the four VDR polymorphisms with coronary artery disease susceptibility.

		Allelic genetic model			Dominant genetic model			Reccesive genetic model			Heterozygote genetic model			Homozygote genetic model		
Subgroup	N	OR[95%CI]	P*/Bon/FDR	I2	OR[95%CI]	P*/Bon/FDR	I2	OR[95%CI]	P*/Bon/FDR	I2	OR[95%CI]	P*/Bon/FDR	I2	OR[95%CI]	P*/Bon/FDR	I2
***rs2228570 Polymorphism***																
**Overall**	11	**1.27 [1.01, 1.59]**	**0.040/0.20/0.04**	**77.00**	**1.42 [1.02, 1.96]**	**0.040/0.20/0.04**	**79.00**	**1.23 [1.01, 1.49]**	**0.030/0.15/0.04**	**29.00**	**1.49 [1.04, 2.13]**	**0.030/0.15/0.04**	**79.00**	**1.34 [1.09, 1.66]**	**0.006/0.03/0.03**	**40.00**
**Race**																
White	7	**1.27 [1.05, 1.54]**	**0.008/0.04/0.0125**	**0.0**	**1.36 [1.06, 1.74]**	**0.010/0.05/0.0125**	**45.0**	**1.33 [1.05, 1.69]**	**0.020/0.10/0.020**	**0.0**	**1.41 [1.08, 1.84]**	**0.010/0.05/0.0125**	**40.0**	**1.42 [1.10, 1.85]**	**0.008/0.04/0.0125**	**0.0**
Asian	3	1.61 [0.96, 2.68]	0.130/0.65/0.16	21.0	2.01 [0.85, 4.75]	0.110/0.55/0.16	90.0	1.33 [0.92, 1.92]	0.130/0.65/0.16	0.0	1.95 [0.76, 5.02]	0.160/0.80/0.16	91.0	**1.60 [1.05, 2.42]**	**0.030/0.15/0.15**	**21.0**
African	1	0.56 [0.35, 0.91]	0.030/0.15/0.05	NA	0.35 [0.12, 1.04]	0.060/0.30/0.06	NA	0.37 [0.17, 0.82]	0.010/0.05/0.05	NA	0.30 [0.09, 1.01]	0.050/0.25/0.06	NA	0.38 [0.17, 0.89]	0.030/0.15/0.05	NA
**HWE#**																
In accordance with HWE	9	**1.33 [1.07, 1.65]**	**0.010/0.05/0.017**	**73.0**	**1.48 [1.06, 2.08]**	**0.020/0.10/0.02**	**79.0**	**1.31 [1.06, 1.62]**	**0.010/0.05/0.017**	**0.0**	**1.55 [1.08, 2.21]**	**0.020/0.10/0.02**	**79.0**	**1.44 [1.14, 1.82]**	**0.002/0.002/0.01**	**0.0**
Departure from HWE	2	1.01 [0.32, 3.14]	0.990/1/0.99	92.0	0.92 [0.16, 5.28]	0.920/1/0.99	88.0	0.77 [0.19, 3.06]	0.710/1/0.99	87.0	1.04 [0.09, 11.86]	0.980/1/0.99	88.0	0.87 [0.18, 4.08]	0.860/1/0.99	88.0
**Sample Size**																
Small(<500)	9	1.17 [0.93, 1.46]	0.19/0.95/0.317	64.00	1.30 [0.98, 1.72]	0.06/0.30/0.15	56.00	1.05 [0.78, 1.39]	0.76/1/0.76	23.00	**1.38 [1.03, 1.84]**	**0.03/0.15/0.15**	**47.00**	1.17 [0.83, 1.64]	0.37/1/0.463	37.00
Large(≥500)	2	1.75 [0.88, 3.47]	0.11/0.55/0.183	94.00	2.18 [0.63, 7.59]	0.22/1/0.275	96.00	**1.58 [1.15, 2.16]**	**0.01/0.05/0.025**	**0.00/0.05/0.025**	**2.07 [0.51, 8.46]**	**0.311/0.31**	**97.00**	**1.82 [1.13, 2.92]**	**0.01/0.05/0.025**	**18.00**
**Genotyping method**																
PCR-RFLP	7	**1.45 [1.11, 1.90]**	**0.01/0.05/0.017**	**75.00**	**1.73 [1.17, 2.55]**	**0.01/0.05/0.017**	**78.00**	1.18 [0.91, 1.55]	0.22/1/0.22	0.00	**1.82 [1.17, 2.82]**	**0.01/0.05/0.017**	**79.00**	**1.41 [1.05, 1.88]**	**0.02/0.10/0.025**	**0.00**
PCR-Taqman	3	0.76 [0.40, 1.47]	0.42/1/0.75	83.00	0.77 [0.41, 1.43]	0.40/1/0.75	66.00	0.83 [0.28, 2.50]	0.75/1/0.75	82.00	0.85 [0.49, 1.50]	0.58/1/0.75	47.00	0.77 [0.25, 2.32]	0.64/1/0.75	80.00
PCR-ABD	1	1.48 [1.00, 2.20]	0.05/0.25/0.1125	NA	1.81 [1.03, 3.20]	0.04/0.20/0.1125	NA	1.43 [0.71, 2.87]	0.32/1/0.32	NA	1.75 [0.95, 3.24]	0.07/0.35/0.1125	NA	1.95 [0.90, 4.22]	0.09/0.45/0.1125	NA
***rs1544410 Polymorphism***																
**Overall**	10	**1.15 [1.09, 1.22]**	**0.00/0.00/0.00**	**13.00**	**1.24 [1.14, 1.36]**	**0.00/0.00/0.00**	**39.00**	**1.16 [1.05, 1.28]**	**0.00/0.00/0.00**	**1.00**	**1.22 [1.11, 1.34]**	**0.00/0.00/0.00**	**57.00**	**1.29 [1.14, 1.46]**	**0.00/0.00/0.00**	**0.00**
**Race**																
White	8	**1.15 [1.08, 1.22]**	**0.000/0.00/0.00**	**29.0**	**1.25 [1.14, 1.37]**	**0.000/0.00/0.00**	**52.0**	**1.15 [1.04, 1.28]**	**0.007/0.035/0.007**	**19.0**	**1.18 [1.06, 1.31]**	**0.002/0.01/0.0025**	**64.0**	**1.30 [1.15, 1.46]**	**0.000/0.00/0.00**	**16.0**
Asian	2	1.21 [0.87, 1.70]	0.260/1/0.5	0.0	1.16 [0.71, 1.88]	0.600/1/0.75	0.0	1.30 [0.79, 2.13]	0.300/1/0.5	0.0	**1.39 [1.12, 1.74]**	**0.003/0.015/0.015**	**0.0**	1.16 [0.46, 2.92]	0.750/1/0.75	0.0
**HWE#**																
In accordance with HWE	6	**1.19 [1.11, 1.28]**	**0.000/0.00/0.00**	**0.0**	**1.33 [1.20, 1.47]**	**0.000/0.00/0.00**	**0.0**	**1.17 [1.04, 1.33]**	**0.010/0.05/0.0125**	**0.0**	1.18 [0.80, 1.74]	0.410/1/0.41	63.0	**1.36 [1.18, 1.57]**	**0.000/0.00/0.00**	**0.0**
Departure from HWE	4	1.05 [0.94, 1.17]	0.400/1/0.5	0.0	1.03 [0.77, 1.38]	0.850	45.0	1.18 [0.86, 1.62]	0.300	25.0	1.21 [1.00, 1.46]	0.050	58.0	1.12 [0.88, 1.41]	0.350	0.0
**Sample Size**																
Small(<500)	4	**1.34 [1.07, 1.68]**	**0.010/0.05/0.017**	**25.0**	1.29 [0.74, 2.25]	0.360/1/0.36	65.0	**1.59 [1.12, 2.24]**	**0.009/0.045/0.017**	**21.0**	1.17 [0.98, 1.41]	0.090/0.45/0.1125	46.0	**1.89 [1.29, 2.79]**	**0.001/0.005/0.005**	**0.0**
Large(≥500)	6	**1.13 [1.06, 1.20]**	**0.000/0.00/0.00**	**0.0**	**1.23 [1.10, 1.37]**	**0.000/0.00/0.00**	**18.0**	1.11 [1.00, 1.23]	0.060/0.30/0.075	0.0	1.21 [0.86, 1.69]	0.270/1/0.27	68.0	**1.24 [1.09, 1.41]**	**0.001/0.005/0.002**	**0.0**
**Genotyping method**																
PCR-RFLP	6	**1.20 [1.00, 1.44]**	**0.05/0.25/0.0625**	**47.00**	1.16 [0.86, 1.57]	0.33/1/0.33	53.00	**1.34 [1.01, 1.77]**	**0.04/0.20/0.0625**	**40.00**	**1.22 [1.07, 1.39]**	**0.00/0.00/0.00**	**31.00**	1.43 [0.99, 2.05]	0.050/0.25/0.0625	39.0
PCR-ABD	4	**1.17 [1.09, 1.26]**	**0.00/0.00/0.00**	**0.00**	1.31 [1.17, 1.45]	0.00**/0.00/0.00**	0.00	1.13 [0.99, 1.28]	0.08/0.4/0.1	0.00	1.26 [0.65, 2.46]	0.50/1/0.5	77.00	**1.31 [1.13, 1.52]**	**0.000/0.00/0.00**	**0.0**
***rs7975232 Polymorphism***																
**Overall**	11	**1.19 [1.04, 1.37]**	**0.01/0.05/0.033**	**70.00**	**1.30 [1.04, 1.62]**	**0.02/0.10/0.00**	**77.00**	**1.06 [0.94, 1.19]**	**0.38/1/0.38**	**10.00**	**1.29 [1.01, 1.66]**	**0.04/0.20/0.05**	**79.00**	**1.18 [1.03, 1.35]**	**0.02/0.10/0.033**	**0.00**
**Race**																
White	8	**1.09 [1.02, 1.17]**	**0.008/0.04/0.013**	**0.0**	**1.17 [1.06, 1.28]**	**0.001/0.005/0.005**	**0.0**	**1.04 [0.92, 1.18]**	**0.560/1/0.56**	**22.0**	**1.18 [1.06, 1.32]**	**0.003/0.015/0.0075**	**12.0**	**1.15 [1.00, 1.32]**	**0.040/0.20/0.05**	**0.0**
Asian	2	1.82 [0.95, 3.49]	0.070/0.35/0.175	84.0	2.45 [0.71, 8.51]	0.160/0.80/0.267	90.0	1.38 [0.77, 2.47]	0.280/1/0.28	0.0	2.35 [0.58, 9.58]	0.230/1/0.28	91.0	1.87 [0.95, 3.67]	0.070/0.35/0.175	0.0
African	1	1.25 [0.59, 2.67]	0.560/1/0.7	NA	1.62 [0.53, 4.90]	0.400/1/0.667	NA	2.53 [0.57, 11.27]	0.220/1/0.667	NA	0.83 [0.27, 2.52]	0.740/1/0.74	NA	2.31 [0.48, 11.26]	0.300/1/0.667	NA
**HWE#**																
In accordance with HWE	9	**1.23 [1.06, 1.43]**	**0.007/0.035/0.015**	**74.0**	**1.38 [1.06, 1.79]**	**0.020/0.100/0.025**	**81.0**	1.10 [0.92, 1.30]	0.290/1/0.29	24.0	**1.40 [1.08, 1.82]**	**0.000/0.00/0.00**	**80.0**	**1.22 [1.05, 1.41]**	**0.009/0.045/0.015**	**0.0**
Departure from HWE	2	0.98 [0.74, 1.31]	0.920/1/1	0.0	1.04 [0.81, 1.34]	0.760/1/1	0.0	0.97 [0.69, 1.35]	0.860/1/1	0.0	0.76 [0.44, 1.31]	0.320/1/1	24.0	1.00 [0.70, 1.43]	1.00/1/1	0.0
**Sample Size**																
Small(<500)	5	**1.10 [0.88, 1.37]**	**0.410/1/0.61**	**0.0**	**1.08 [0.86, 1.35]**	**0.490/1/0.61**	**0.0**	1.08 [0.80, 1.45]	0.610**/1/0.61**	0.0	**0.88 [0.62, 1.24]**	**0.460/1/0.61**	**0.0**	1.10 [0.80, 1.52]	0.550**/1/0.61**	0.0
Large(≥500)	6	**1.22 [1.03, 1.45]**	**0.020/0.10/0.033**	**84.0**	**1.40 [1.03, 1.90]**	**0.030/0.15/0.037**	**88.0**	1.05 [0.92, 1.20]	0.460/1/0.46	29.0	**1.46 [1.09, 1.96]**	**0.010/0.05/0.033**	**87.0**	**1.20 [1.03, 1.39]**	**0.020/0.10/0.033**	**12.0**
**Genotyping method**																
PCR-RFLP	3	1.35 [0.69, 2.64]	0.39/1/0.70	90.00	1.65 [0.60, 4.52]	0.33/1/0.70	94.00	0.99 [0.72, 1.36]	0.94/1/0.94	0.00	1.51 [0.37, 6.21]	0.56/1/0.70	95.00	1.16 [0.70, 1.90]	0.56/1/0.70	41.0
PCR-Taqman	3	1.07 [0.89, 1.29]	0.48/1/0.67	0.00	0.91 [0.60, 1.39]	0.67/1/0.67	0.00	**3.02 [1.31, 6.96]**	**0.01/0.05/0.05**	**0.00**	1.08 [0.82, 1.42]	0.60/1/0.67	0.00	**2.51 [1.05, 5.99]**	**0.04/0.20/0.10**	**0.0**
PCR-ABD	5	**1.11 [1.03, 1.19]**	**0.00/0.00/0.00**	**0.00**	**1.22 [1.10, 1.35]**	**0.00/0.00/0.00**	**0.00**	1.04 [0.91, 1.19]	0.60/1/0.60	0.00	**1.24 [1.11, 1.38]**	**0.00/0.00/0.00**	**0.00**	**1.17 [1.01, 1.36]**	**0.04/0.2/0.05**	**0.0**
***rs731236 Polymorphism***																
**Overall**	9	**0.93 [0.88, 0.99]**	**0.03/0.15/0.067**	**4.00**	0.93 [0.84, 1.02]	0.12/0.60/0.15	7.00	**0.90 [0.81, 1.00]**	**0.04/0.20/0.067**	**0.00**	0.90 [0.81, 1.00]	0.38/1/0.38	1.00	**0.87 [0.77, 0.99]**	**0.03/0.15/0.067**	**0.00**
**Race**																
White	6	**0.93 [0.87, 1.00]**	**0.040/0.20/0.117**	**10.0**	**0.92 [0.83, 1.02]**	**0.100/0.50/0.125**	**12.0**	**0.90 [0.81, 1.01]**	**0.070/0.35/0.1167**	**0.0**	**0.94 [0.84, 1.05]**	**0.280/1/0.28**	**10.0**	**0.87 [0.77, 1.00]**	**0.050/0.25/0.1167**	**0.0**
Asian	2	0.94 [0.74, 1.18]	0.590/1/0.89	63.0	1.02 [0.74, 1.42]	0.890/1/0.89	58.0	0.78 [0.51, 1.20]	0.260/1/0.89	33.0	1.12 [0.79, 1.59]	0.540/1/0.89	35.0	0.93 [0.40, 2.13]	0.860/1/0.89	64.0
African	1	0.91 [0.60, 1.38]	0.650/1/0.76	NA	0.78 [0.31, 1.96]	0.600/1/0.76	NA	0.92 [0.53, 1.59]	0.760/1/0.76	NA	0.80 [0.30, 2.11]	0.650/1/0.76	NA	0.77 [0.29, 2.00]	0.590/1/0.76	NA
**HWE#**																
In accordance with HWE	7	0.93 [0.87, 1.00]	0.06/0.30/0.10	16.00	0.92 [0.81, 1.05]	0.24/1/0.30	29.00	0.90 [0.81, 1.01]	0.06/0.30/0.10	0.00	0.95 [0.83, 1.09]	0.48/1/0.48	23.00	**0.88 [0.77, 1.00]**	**0.05/0.25/0.10**	**2.00**
Departure from HWE	2	0.90 [0.70, 1.14]	0.37/1/0.73	4.00	0.92 [0.66, 1.26]	0.59/1/0.73	0.00	0.85 [0.46, 1.55]	0.59/1/0.73	50.00	0.95 [0.65, 1.37]	0.77/1/0.77	0.00	0.82 [0.47, 1.44]	0.50/1/0.73	36.00
**Sample Size**																
Small(<500)	3	1.07 [0.84, 1.38]	0.57/1/0.81	0.00	1.15 [0.77, 1.71]	0.51/1/0.81	0.00	1.05 [0.73, 1.50]	0.81/1/0.81	0.00	1.10 [0.64, 1.88]	0.73/1/0.81	12.00	1.13 [0.71, 1.77]	0.61/1/0.81	0.00
Large(≥500)	6	**0.92 [0.86, 0.99]**	**0.02/0.10/0.05**	**13.00**	0.91 [0.82, 1.01]	0.09/0.45/0.1125	11.00	**0.89 [0.79, 0.99]**	**0.03/0.15/0.05**	**0.00**	0.95 [0.84, 1.06]	0.33/1/0.33	7.00	**0.85 [0.74, 0.97]**	**0.02/0.10/0.05**	**7.00**
**Genotyping method**																
PCR-RFLP	2	0.90 [0.70, 1.14]	0.37/1/0.74	4.00	0.92 [0.66, 1.26]	0.59/1/0.74	0.00	0.85 [0.46, 1.55]	0.59/1/0.74	50.00	0.95 [0.65, 1.37]	0.77/1/0.74	0.00	0.82 [0.47, 1.44]	0.50/1/0.74	36.00
PCR-Taqman	2	0.92 [0.77, 1.09]	0.32/1/0.533	0.00	0.97 [0.72, 1.30]	0.83/1/0.83	0.00	0.83 [0.63, 1.09]	0.18/0.9/0.533	0.00	1.05 [0.76, 1.44]	0.77/1/0.83	0.00	0.84 [0.59, 1.19]	0.32/1/0.533	0.00
PCR-ABD	5	0.93 [0.84, 1.03]	0.18/0.9/0.3	44.00	0.92 [0.77, 1.09]	0.31/1/0.388	51.00	0.92 [0.82, 1.03]	0.15/0.75/0.3	0.00	0.94 [0.79, 1.11]	0.46/1/0.46	44.00	0.88 [0.73, 1.05]	0.16/0.80/0.30	33.00

OR = odd ratio; CI = Confidence Interval; HB = Hospital Based; PB = Population Based; # P value for Hardy–Weinberg equilibrium test in controls; * P value for meta-analysis, Bon = p value in Bonferroni test; FDR = false discovery rate. PCR-RFLP = polymerase chain reaction-restriction fragment length polymorphism

PCR-Taqman = polymerase chain reaction with Taqman probe; PCR-ABD = polymerase chain reaction using Assay by Design (ABD) kits from Applied Biosystems (Carlsbad, CA, USA).

Significant results are marked in bold.

### rs2228570 polymorphism and CAD susceptibility

**Fig 1:**
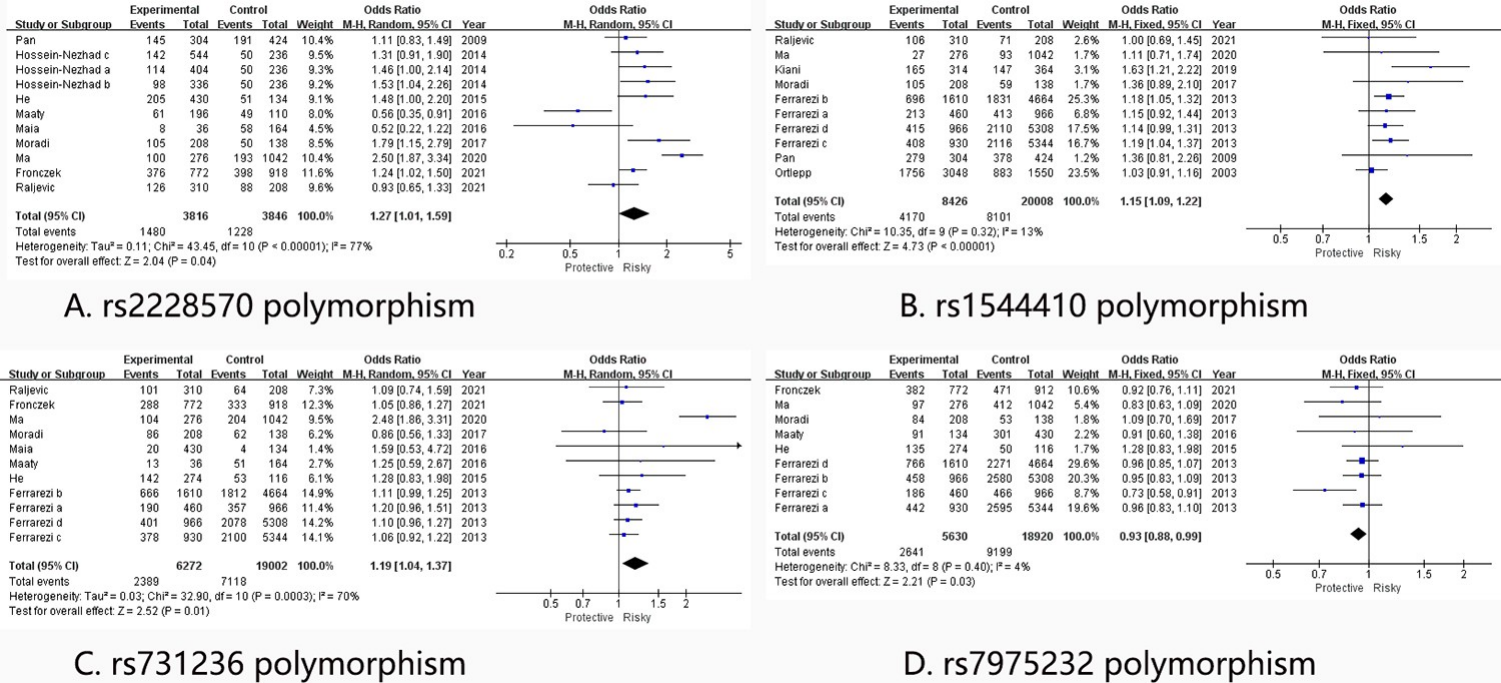
Forest plot of CAD risk associated with the VDR polymorphism. CAD = Coronary artery disease, A: rs2228570 polymorphism; B: rs1544410 polymorphism; C: rs731236 polymorphism; D: rs7975232 polymorphism. VDR = vitamin D receptor, OR = odd ration, CI = confidence interval.

**Fig 2:**
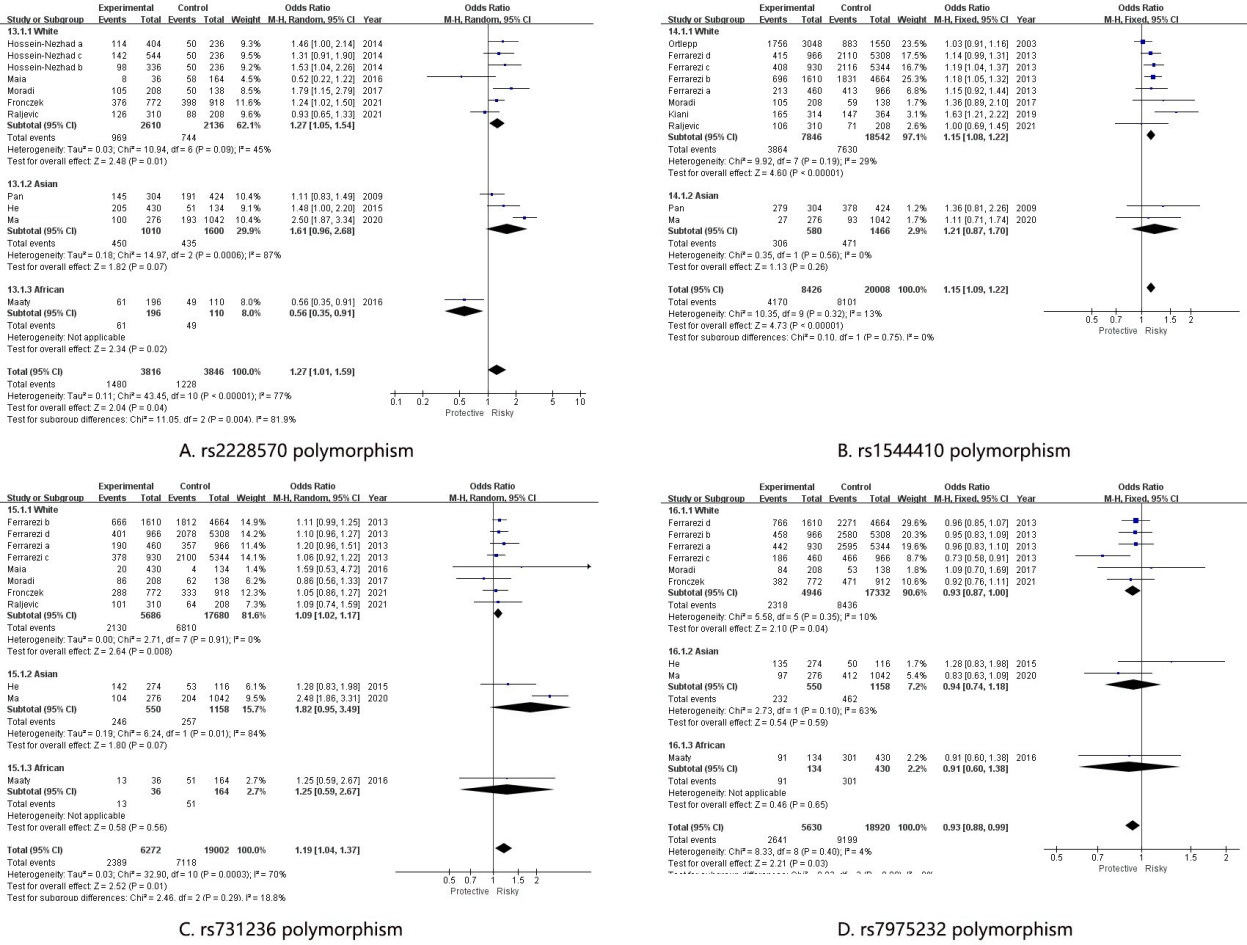
Forest plot of CAD risk associated with the VDR polymorphism in the subgroup analysis stratified by race. A: rs2228570 polymorphism; B: rs1544410 polymorphism; C: rs731236 polymorphism; D: rs7975232 polymorphism. CAD = Coronary artery disease, VDR = vitamin D receptor, OR = odd ration, CI = confidence interval.

For the rs2228570 polymorphism, the pooled results showed significant associations with high heterogeneity in all genetic model: allelic genetic model (OR[95%CI] = 1.27 [1.01, 1.59], I2 = 77%) (Fig 1A), dominant genetic model (OR[95%CI] = 1.42 [1.02, 1.96], I2 = 79%), recessive genetic model (OR[95%CI] = 1.23 [1.01, 1.49], I2 = 29%), heterozygote genetic model (OR[95%CI] = 1.49 [1.04, 2.13], I2 = 79%) and homozygote genetic model (OR[95%CI] = 1.34 [1.09, 1.66], I2 = 40%). In order to analyze the high heterogeneity, four subgroup analyses based on Race, HWE, Sample size and Genotyping method were conducted. In the subgroup analysis stratified by race, high heterogeneity were significantly reduced in the White subgroup in all genetic models: allelic genetic model (OR[95%CI] = 1.27 [1.05, 1.54], I2 = 45%) Fig 2A, dominant genetic model (OR[95%CI] = 1.36 [1.06, 1.74], I2 = 45%), recessive genetic model (OR[95%CI] = 1.33 [1.05, 1.69], I2 = 0%), heterozygote genetic model (OR[95%CI] = 1.41 [1.08, 1.84], I2 = 40%) and homozygote genetic model (OR[95%CI] = 1.42 [1.10, 1.85], I2 = 0%); significant association was only detected in homozygote genetic model (OR[95%CI] = 1.60 [1.05, 2.42], I2 = 21%) in Asian; no association was observed in African. As for the subgroup analyses based on HWE, Sample size and Genotyping method, significant associations with high heterogeneity were also observed, which indicated these results should be interpreted with cautions.

### rs1544410 polymorphism and CAD susceptibility

For the rs1544410 polymorphism, significant associations with low heterogeneity were discovered in both pooled and subgroup analyses. From the pooled results, increased risk of CAD were observed in all genetic models: allelic genetic model (OR[95%CI] = 1.15 [1.09, 1.22], I2 = 13%) Fig 1B, dominant genetic model (OR[95%CI] = 1.24 [1.14, 1.36], I2 = 39%), recessive genetic model (OR[95%CI] = 1.16 [1.05, 1.28], I2 = 1%), heterozygote genetic model (OR[95%CI] = 1.22[1.11, 1.34], I2 = 57%) and homozygote genetic model (OR[95%CI] = 1.29 [1.14, 1.46], I2 = 0%). In the White population, increased risk of CAD were also observed in all genetic models: allelic genetic model (OR[95%CI] = 1.15 [1.08, 1.22], I2 = 29%) Fig 2B, dominant genetic model (OR[95%CI] = 1.25[1.14, 1.37], I2 = 52%), recessive genetic model (OR[95%CI] = 1.15 [1.04, 1.28], I2 = 19%), heterozygote genetic model (OR[95%CI] = 1.18[1.06, 1.31], I2 = 64%) and homozygote genetic model (OR[95%CI] = 1.30 [1.15, 1.46], I2 = 16%). However, increased risk of CAD was only observed in the heterozygote model (OR [95%CI] = 1.39 [1.12, 1.74], I2 = 0%) in Asian. In subgroup analyses stratified by HWE, increased risks were observed in allelic (OR [95%CI] = 1.19 [1.11, 1.28], I2 = 0%), dominant (OR [95%CI] = 1.33 [1.20, 1.47], I2 = 0%), recessive (OR [95%CI] = 1.17 [1.04, 1.33], I2 = 0%) and homozygote (OR [95%CI] = 1.36 [1.18, 1.57], I2 = 0%) genetic models in subgroup in accordance with HWE. For subgroup analyses based on samples size, increased risks were widely observed in both Large subgroup (allelic (OR [95%CI] = 1.13 [1.06, 1.20], I2 = 0%); dominant (OR [95%CI] = 1.23 [1.10, 1.37], I2 = 18%); homozygote (OR [95%CI] = 1.24 [1.09, 1.41], I2 = 0%)) and Small subgroup (allelic (OR [95%CI] = 1.34 [1.07, 1.68], I2 = 25%); recessive (OR [95%CI] = 1.59 [1.12, 2.24], I2 = 21%); homozygote (OR [95%CI] = 1.89 [1.29, 2.79], I2 = 0%)). As for the subgroup analyses based on genotyping method, although significant associations were also widely detected, increased heterogeneity should not be neglected.

### rs731236 polymorphism and CAD susceptibility

Same as the rs2228570 polymorphism, significant associations with high heterogeneity were widely observed in overall and subgroup analysis. In the overall analysis, increased risk in allelic (OR [95%CI] = 1.19 [1.04, 1.37], I2 = 70%) (Fig 1C), dominant (OR [95%CI] = 1.30 [1.04, 1.62], I2 = 77%), heterozygote (OR [95%CI] = 1.29 [1.01, 1.66], I2 = 79%) and homozygote (OR [95%CI] = 1.18 [1.03, 1.35], I2 = 0%) genetic models were discovered. Reduced heterogeneity with increased CAD risks were detected in the White population in allelic (OR [95%CI] = 1.09 [1.02, 1.17], I2 = 0%) (Fig 2C), dominant (OR [95%CI] = 1.17 [1.06, 1.28], I2 = 0%), heterozygote (OR [95%CI] = 1.18 [1.06, 1.32], I2 = 12%) and homozygote (OR [95%CI] = 1.15 [1.00, 1.32], I2 = 0%) genetic models, however, no associations were observed in both Asian and African. As for the subgroup analyses based on HWE, Sample size and Genotyping method, wide significant associations with unsolved heterogeneity were observed.

### rs7975232 polymorphism and CAD susceptibility

Interestingly, decreased risks of CAD were firstly discovered in overall analysis and subgroup analysis based on Race and Sample size. In overall analysis, decreased CAD risks were detected in allelic (OR [95%CI] = 0.93[0.88, 1.00], I2 = 4%) (Fig 1D), heterozygote (OR [95%CI] = 0.90 [0.81, 1.00], I2 = 0%) and homozygote (OR [95%CI] = 0.87 [0.77, 0.99], I2 = 0%) genetic models. In the White population, decreased CAD risks were observed in allelic (OR [95%CI] = 0.93 [0.87, 1.00], I2 = 10%) (Fig 2D) and homozygote (OR[95%CI] = 0.87 [0.77, 1.00], I2 = 0%) genetic models. Decreased CAD risks in allelic (OR [95%CI] = 0.92 [0.86, 0.99], I2 = 13%), recessive (OR [95%CI] = 0.89 [0.79, 0.99], I2 = 0%) and homozygote (OR [95%CI] = 0.85 [0.74, 0.97], I2 = 7%) genetic models were observed in Large subgroup. Although decreased risks were remarkably observed in rs7975232 polymorphism, more studies were required to validate the decreased association.

### Sensitivity analysis of associations between VDR polymorphisms and CAD susceptibility

**Fig 3:**
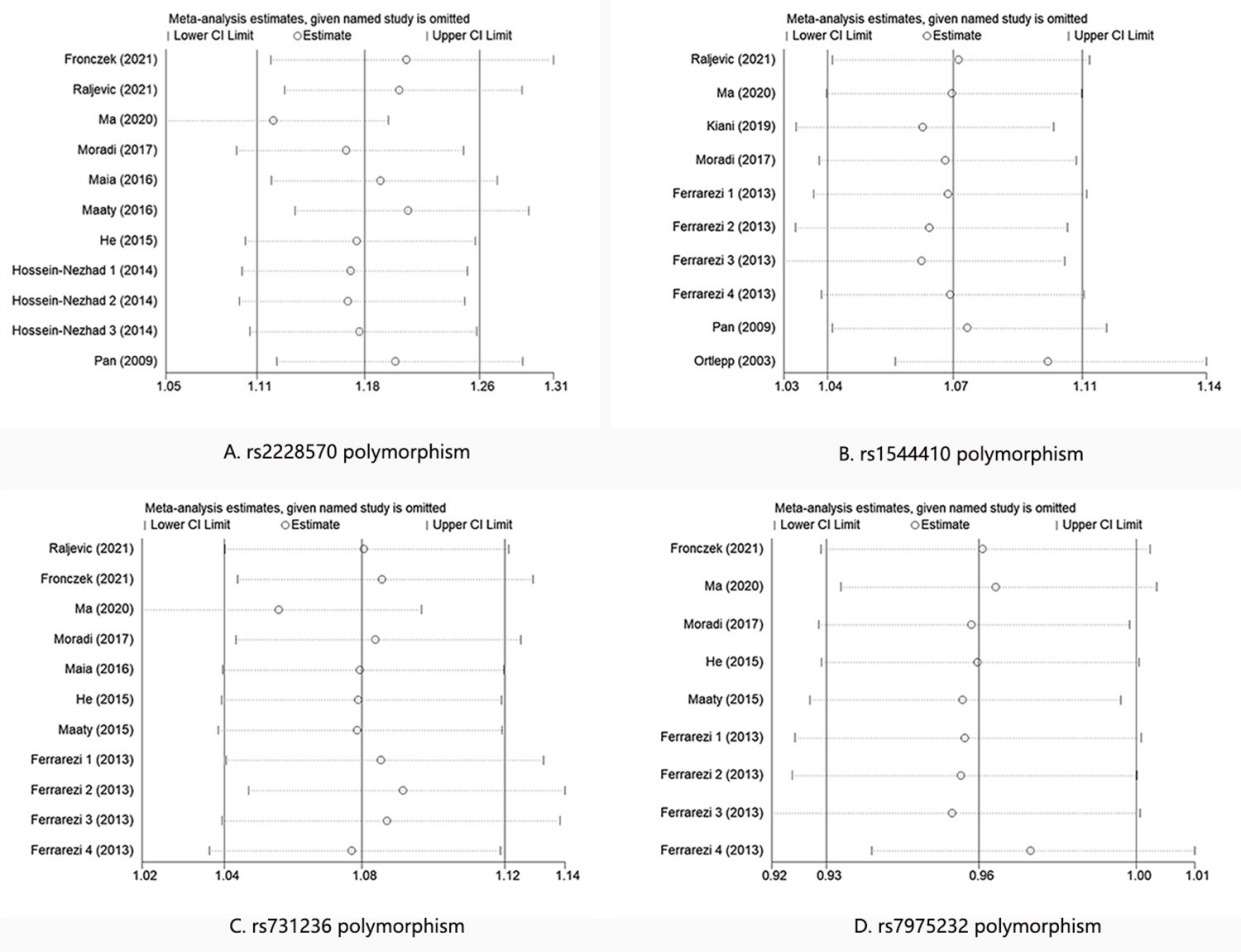
Sensitivity analysis of CAD risk associated with the VDR polymorphism. A: rs2228570 polymorphism; B: rs1544410 polymorphism; C: rs731236 polymorphism; D: rs7975232 polymorphism. CAD = Coronary artery disease, VDR = vitamin D receptor, OR = odd ration, CI = confidence interval.

We conducted the sensitive analyses on VDR polymorphism and CAD risk by omitting one study at a time in the calculation of the summary outcome (Fig 3). The results showed that no single study fundamentally changed the associations between these four VDR polymorphisms and CAD risk, which indicated that our meta-analysis results were relatively stable.

### Publication bias

**Fig 4:**
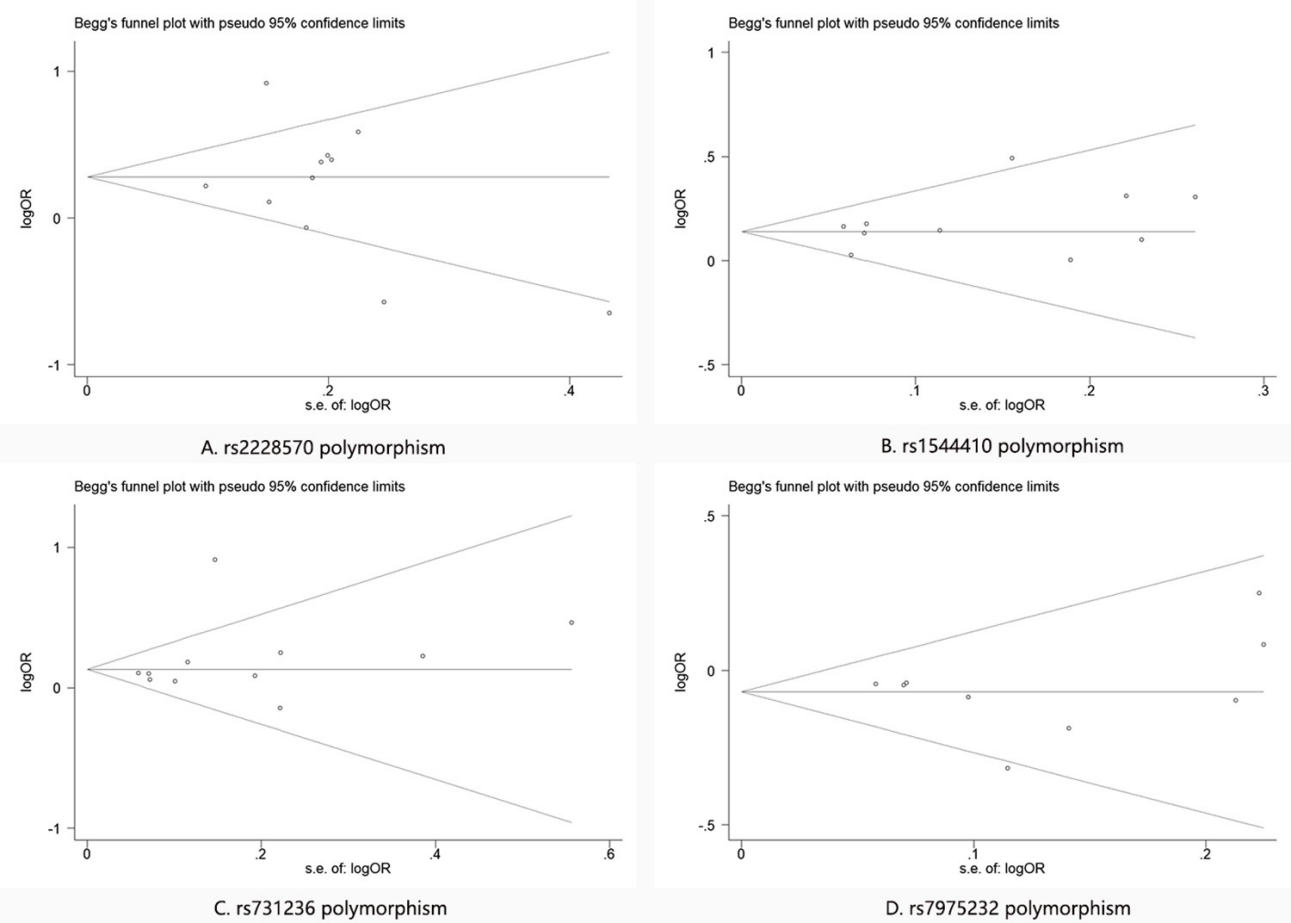
The Begg’s plot of Publication bias for the VDR polymorphism. A: rs2228570 polymorphism; B: rs1544410 polymorphism; C: rs731236 polymorphism; D: rs7975232 polymorphism. CAD = Coronary artery disease, VDR = vitamin D receptor, OR = odd ration, CI = confidence interval.

The Egger’s test was introduced to analyze the publication bias, the P value for the test of these four VDR polymorphisms were 0.423 (rs2228570), 0.218 (rs1544410), 0.396 (rs731236) and 0.980 (rs7975232), respectively. Moreover, the Begg’s funnel plots of these four polymorphisms were symmetrical (Fig 4). The results based on the Egger’s test and the Begg’s funnel plots indicated no publication bias for these four VDR polymorphisms with CAD risk.

### Trial sequential analysis of associations between VDR polymorphisms and CAD susceptibility

**Fig 5:**
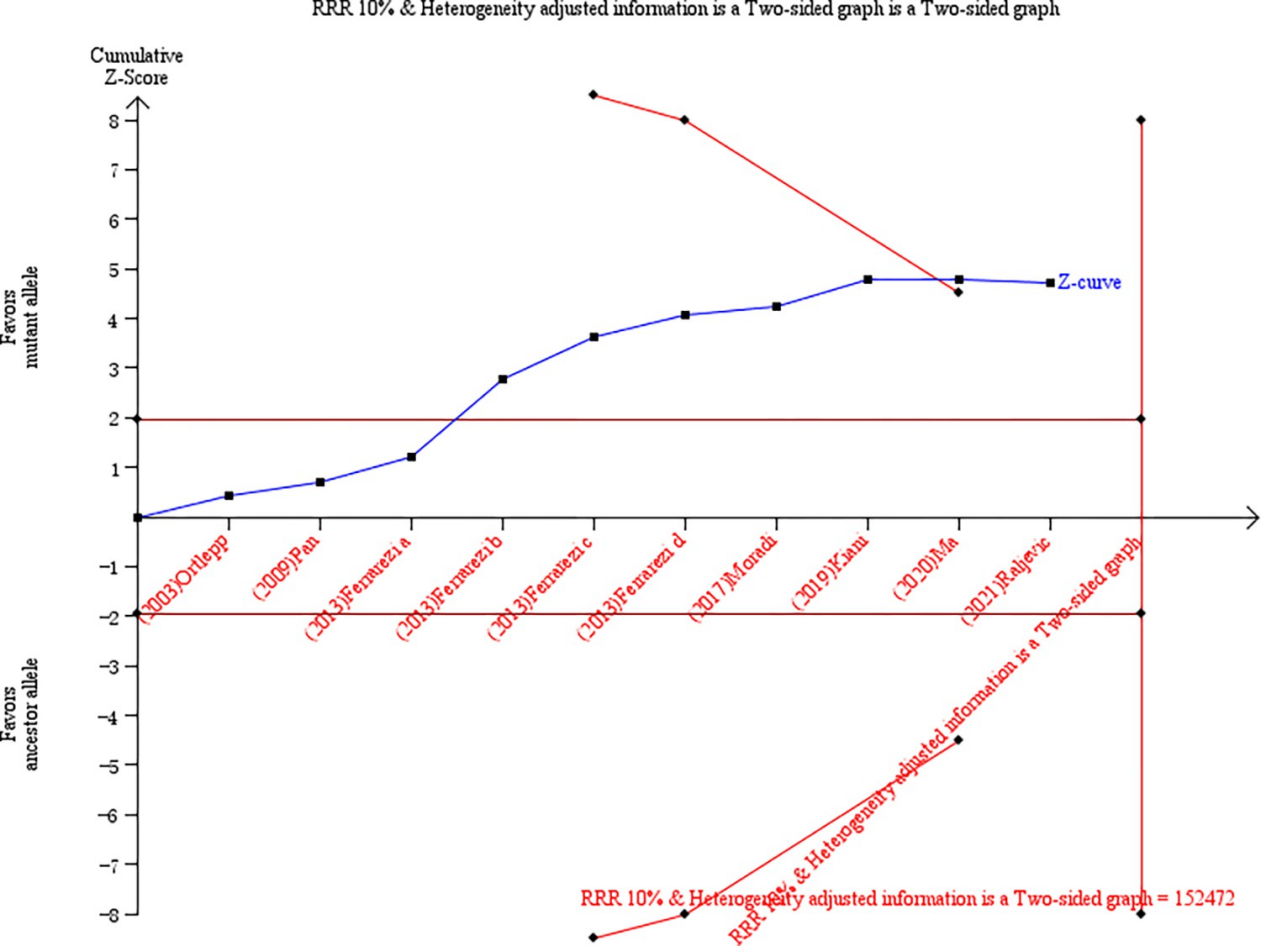
Trial sequential analysis of VDR rs1544410 polymorphism in overall population. VDR = vitamin D receptor.

**Fig 6:**
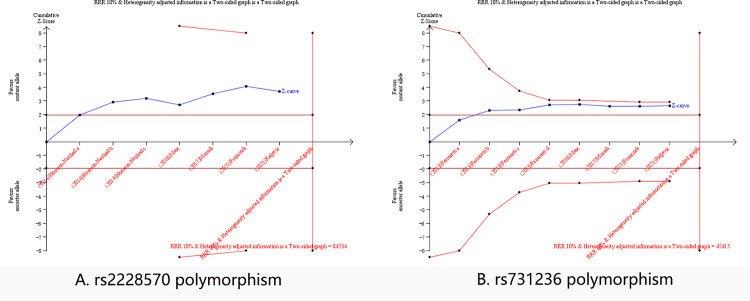
Trial sequential analysis of VDR rs2228570 and rs731236 polymorphisms in the White population. VDR = vitamin D receptor.

Based on our analysis, increased CAD risks with low heterogeneity in the overall analysis of the rs1544410 polymorphism and the White population of the rs2228570 and rs731236 polymorphisms were discovered. Therefore, a trial sequential analysis was introduced to validate that our discoveries above were not false positive results. The allelic genetic model is a natural model of inheritance with a stronger genotype-phenotype association, which also does not pre-assume any interactions between the numbers of variant alleles. Therefore, we chose the allelic genetic model of the rs1544410 polymorphism in overall population and the rs2228570 and rs731236 polymorphism in the White population to conduct the trial sequential analysis. The X and Y axes represent the number of patients and the cumulative Z score, respectively. Within the designed assumptions of confidence and effect size, the information size for the rs1544410 polymorphism are 152472, the Z curves not only cross the statistical significance line (Z = 1.96, P = 0.05), but also cross the O’ Brien Fleming boundaries (Fig 5), indicating that the significance level of our study was a true positive result. However, for the rs2228570 and rs731236 polymorphisms, although the Z curves cross the statistical significance line (Z = 1.96, P = 0.05), but not cross the O’ Brien Fleming boundaries, which indicated more studies were required, and the information size for the rs2228570 and rs731236 polymorphisms were 84534 and 42415 respectively (Fig 6).

### Bioinformatics analysis

**Fig 7:**
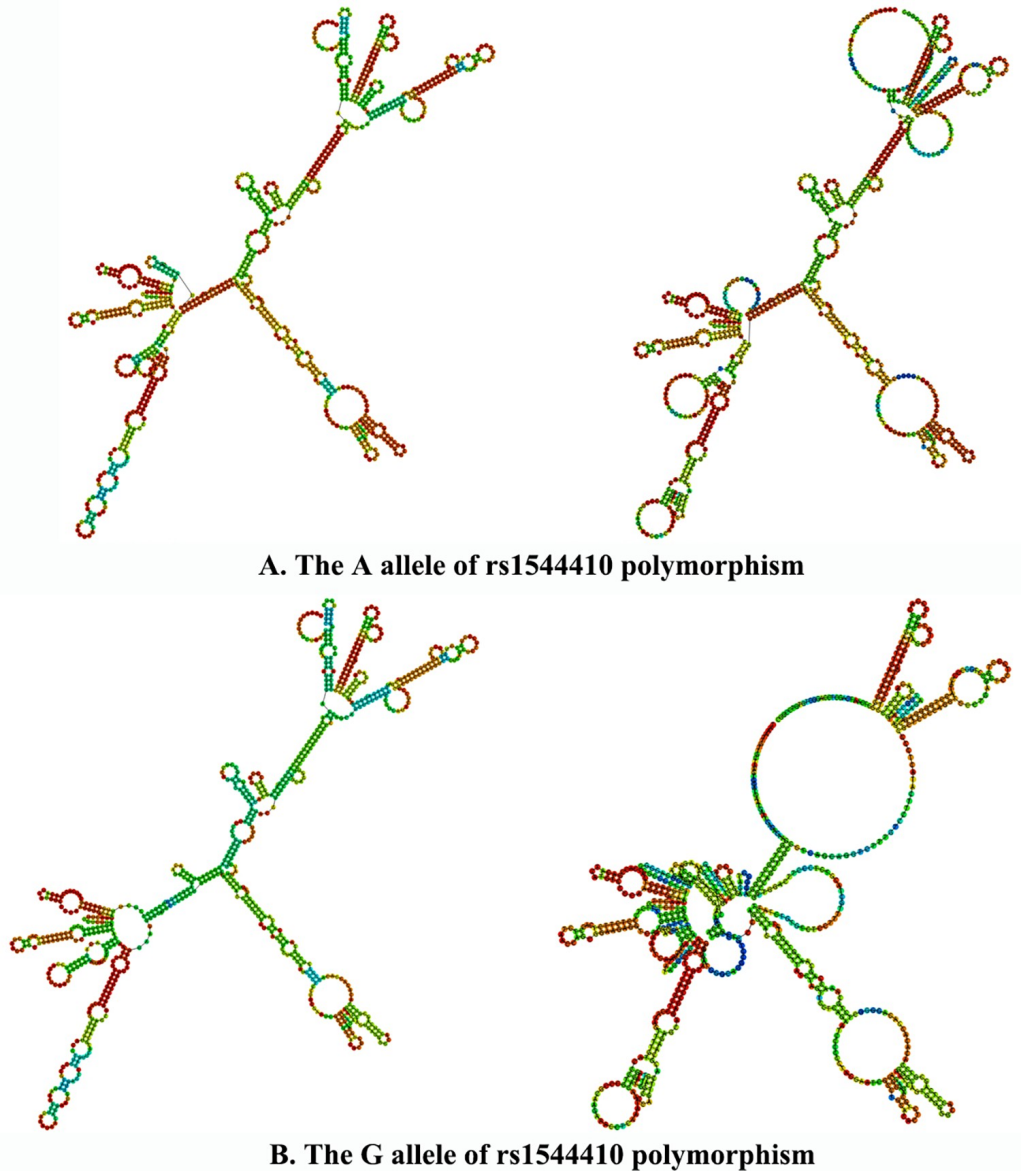
The RNAfold structure analysis of the VDR rs1544410 polymorphism. A: rs2228570 polymorphism; B: rs731236 polymorphism. VDR = vitamin D receptor.

Based on the genomic context obtained from the Ensembl database, the VDR rs2228570 polymorphism caused a “start lost”, the rs1544410 and rs7975232 polymorphisms were intron variants, the rs731236 polymorphism was the synonymous variant. Hence, we analyzed the sequences of the four polymorphisms and the results from the SNPinfo database showed the VDR rs2228570 and rs731236 polymorphism were predicted the function of Splicing (Table 3). In addition, the secondary structure of DNA at the VDR rs1544410 sequences was predicted using RNAfold. The minimum free energy (MFE) and the free energy of the thermodynamic ensemble (FETE) of the rs1544410 polymorphism were -264.30 kcal/mol and -276.99 kcal/mol for the wild A allele, -265.80 kcal/mol and -278.74 kcal/mol for the mutant G allele, respectively. Based on the predicted free energy of the rs1544410 polymorphisms, the secondary structure of the polymorphisms was determined. Compared to the wild allele, the mutant alleles of the rs1544410 polymorphism caused a structure change which was pointed with arrows in Fig 7.

**Table 3:** The potential function of the VDR polymorphisms predicted by SNPinfo.

rs	Position	Allele	TFBS	Splicing (site)	Splicing (ESE or ESS)	Splicing (abolish domain)	miRNA (miRanda)	miRNA (Sanger)	nsSNP	Stop Codon	Polyphen	SNPs3D (svm profile)	SNPs3D (svm structure)	RegPotential	Conservation	Distance (bp)
rs2228570	46559162	G/A	--	--	Y	--	--	--	Y	--	--	1	0	0.264195	1	37575||25919
rs1544410	46526102	T/C	--	--	--	--	--	--	--	--	--	--	--	0.083913	0	4515||58979
rs7975232	46525104	A/C	--	--	--	--	--	--	--	--	--	--	--	0.089729	0	3517||59977
rs731236	46525024	G/A	--	--	Y	--	--	--	--	--	--	--	--	0.516109	0.175	3437||60057

## Discussion

Coronary artery disease (CAD) is a disease with very high morbidity and mortality. Early prevention based on genetic polymorphism can reduce the incidence of CAD [37, 38]. In our study, four common single nucleotide polymorphisms (SNPs) in vitamin D receptor (VDR) gene (rs2228570, rs1544410, rs731236 and rs7975232) were comprehensively analyzed and subgroups analysis based on race, samples size, genetic features were performed. There was no genome-wide association study regarding the associations between the VDR polymorphisms and CAD susceptibility, and the four common VDR polymorphisms were widely discussed with inconsistent results, therefore, we chose these four common VDR polymorphisms to investigate the associations between the four VDR polymorphisms and coronary artery disease (CAD) susceptibility.

In the previous meta-analysis [39–41], increased risks were found both in rs1544410 and rs731236 polymorphism, which agreed with our results, but decreased risk of rs2228570 was observed in his study. After careful analysis, we supposed the small sample size and different data recruiting methods could contribute to the discrepancy. In the data retrieving of two included studies (Ferrarezil et al. [35] and Nezhad et al. [34]), the author pooled different small group based on severity of CAD into one group, which caused extremely high heterogeneity. In our analysis, we extracted each small group as one single study to reduce the heterogeneity and positive results with no or subtle heterogeneity were widely observed. Jiang L reported a dose-response meta-analysis based on full subgroups stratified by sex, age, race, et al. and found prospective evidence for further testing of the utility of ferritin levels in predicting T2D risk in a sex-specific manner [42, 43], therefore, we completed exhaustive subgroup analysis stratified by race, HWE, sample size and genotyping method to explore the source of heterogeneity and the potential associations in subgroup, and many interesting results were discovered.

In the overall analysis, the rs1544410 polymorphism was discovered to be associated with an increased risk of CAD in all five genetic models and the positive results were verified by TSA in the allelic genetic model, which indicate that the role of the rs1544410 polymorphism in the VDR gene as a risk factor for CAD. For the mutant b allele, it has a 15% increased CAD risk compared to the B allele. In terms of genotype, the Bb and bb genotype have 22% and 29% increased CAD risk compared to the BB genotype, respectively. As for the rs2228570 and rs731236 polymorphism, increased risk with high heterogeneity were widely observed, the mutant f allele has a 27% increased risk compared to the F allele for the rs2228570 polymorphism; for the rs731236 polymorphism, the mutant t allele has a 19% increased risk compared to the T allele. Interesting findings emerged on the rs7975232 polymorphism, decreased risks were firstly observed. The mutant a allele has a 7% decreased CAD risk compared to the A allele, and the aa genotype has a 13% decreased CAD risk compared to the AA genotype, however, the relative small sample size could have an influence on the evaluation of the rs7975232 polymorphism, and more well-designed studies were required to solidate the potential protective role of the rs7975232 polymorphism.

In the subgroup analysis, the high heterogeneity of the rs2228570 and rs731236 polymorphisms were significantly reduced in the White population. In the White population, increased CAD risks were extensively detected in the rs1544410, rs2228570 and rs731236 polymorphism. However, in Asian subgroup, an 60% increased CAD risk of the ff genotype is observed in the rs2228570 polymorphism compared to the Asian FF genotype, and no association is detected in the rs1544410 and rs731236 polymorphisms. The results in subgroup analysis stratified by race may indicate the White population with rs2228570, rs1544410 and rs731236 are more susceptible to CAD. As for the rs7975232 polymorphism, in the White population, the mutant a allele has a 7% decreased CAD risk compared to the A allele. Sample size is an important parameter in the case-control studies. In the subgroup analysis based on sample size, we detected that increased or decreased risks of the four VDR polymorphisms were widely observed in the large subgroup, which implied case-control studies with sufficient sample size could discover more meaningful data. Homogeneity is a crucial factor in the statistical Analysis, therefore HWE and genotyping typing method were analyzed in different subgroups, however, the results showed these two-subgroup analysis did not seem to affect the high heterogeneity. Compared to the traditional risk factors like smoking, being overweight, and lack of exercise et al., the VDR polymorphisms associated with CAD susceptibility we found could help the population identify CAD earlier and provide individualized treatment.

Lower plasma level of vitamin D was associated with increased risk of CAD [6, 44–46]. VDR is the crucial signal transduction molecule in the vitamin D pathway. From animal research reported by Xiang et al. [47], overexpressing the vitamin D receptor could inhibit the formation of atherosclerotic plague in APOE-deficient mice. In the CAD population, the TT genotype of rs2228570 polymorphism had a lower serum level of vitamin D compared to CC genotype [29]; for rs1544410 and rs731236 polymorphisms, the mutant genotype was associated with the lower plasma level of vitamin D [34, 35]. The polymorphisms in VDR may have an influence in the interaction between VDR and Vitamin D and the serum level of Vitamin D. Causal inference analysis analyze the functional polymorphisms in a gene whether can causally trigger the development of a related disease through mediating the expression of this gene in specific tissues [48, 49]. Zhang F et al. reported the genetically determined PTSD confers a causal effect on depression and depressed affect, but not major depressive disorder [50], moreover, deep learning or machine learning is a hot topic in classification and prediction of diseases based on biomarkers [51, 52], which inspired us to conduct the causal inference analysis of the functional VDR polymorphisms in CAD and discuss the possibility to use the vitamin D receptor genetic variants related to CHD for the prediction or early diagnosis of CHD in our next mechanism study.

The VDR rs2228570 polymorphism caused a “start lost”, the rs1544410 and rs7975232 polymorphisms were intron variant, the rs731236 polymorphism was the synonymous variant. The VDR rs2228570 and rs731236 polymorphism were predicted the function of Splicing. In addition, the secondary structure of DNA at the VDR rs1544410 sequences was predicted by using the RNAfold, which indicated that the mutant G allele could cause an easier dispensation from the DNA double helix structure. The SNP in rs2228570 polymorphism is located in the exon 2, which is near the translation start sequence, and the mutant T allele causes a structural modification of three amino acids longer protein leading to the change of potential protein function [53]. Unlike the rs2228570 polymorphism, the rs1544410, rs7975232 and rs731236 polymorphisms are located near the 3’ end of the gene and cause no structural transformation [53], but they have a strong linkage disequilibrium (LD) [54]. The AAC haplotype composed by the A allele of rs1544410, A allele of rs7975232 and C allele of rs731236 was associated with an increased risk of CAD in type 2 diabetes subjects reported by Ferrarezi et al. [35], furthermore, a VDR GATG haplotype (G allele of rs731236, A allele of rs7975232, T allele of rs1544410 and G allele of rs2228570) was found to be associated with atherosclerotic disease in rheumatoid arthritis patients [55]. These studies suggest a joint role of the three polymorphisms in CAD susceptibility. Besides, acetyl-cytidine on RNA expression is also playing key role on the human diseases. Gehui Jin et al. reported the role and mechanism of ac4C in gene-expression regulation and demonstrated the relevance of ac4C to a variety of human diseases [56]. We found the changed RNA second structure in mutant allele of the VDR polymorphism, the changed structure may provide potential acetyl-cytidine loci and affect the RNA expression, which provide direction for our next mechanistic studies.

There were several limitations in our meta-analysis. Firstly, the language was restricted to English and Chinese, which will cause the limited number of studies included; secondly, other unknown polymorphisms in VDR polymorphisms could also be associated the CAD susceptibility, more case-control studies with comprehensive clinical outcomes and GWAS studies were required; thirdly, the rs1544410, rs7975232 and rs731236 polymorphism are in strong LD, haploid factors with CAD risk need to be considered; fourthly, the mechanisms of the VDR polymorphism on the VDR gene or RNA or protein were not discussed enough, further mechanistic studies are required; at last, genetic factor was the one side for CAD risk, the interaction between environmental risk factors should be considered.

## Conclusion

Our analysis supports the role of the rs1544410 polymorphism in the VDR gene as a risk factor for CAD. The VDR rs2228570 and rs731236 polymorphisms were associated with increased CAD risks in the White population. Restrict decreased CAD risk was firstly discovered in the rs7975232 polymorphism.

## Supporting information

S1 Table The PRISMA flow diagram. (DOCX)

S2 Table Scale for quality assessment. (DOCX)

S3 Table The PRISMA 2009 checklist. (DOCX)

## List of abbreviations

VDR: vitamin D receptor
CAD: coronary artery disease
ORs: odds ratios
TSA: trial sequential analysis
