# Supplementary material for: Four common vitamin D receptor polymorphisms and coronary artery disease susceptibility: A trial sequential analysis
Source: PLoS One. 2022 Oct 3;17(10):e0275368. doi: 10.1371/journal.pone.0275368 (PMC9529108; doi:10.1371/journal.pone.0275368)
Supplement: S1 Table — (DOCX) [file pone.0275368.s001.docx]

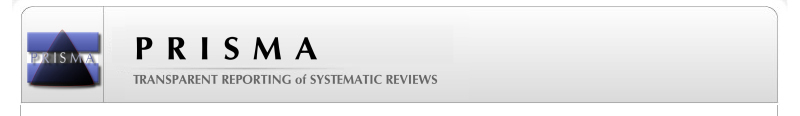
**PRISMA 2009 Flow Diagram**

Articles included in quantitative synthesis (meta-analysis)
(n = 13)

(11 studies for FokI polymorphism; 10 studies for BsmI and 11 for TaqI polymorphisms; 9 studies for ApaI polymorphism)

## Included

Articles included in qualitative synthesis
(n = 17)

Full-text articles excluded,

Lack of detailed genotype distribution data
(n = 4)

Title and abstract excluded
(n = 14)

Title and abstract screened
(n = 57)

Full-text articles excluded,

Reviews, editorial

(n = 36)

Full-text articles assessed for eligibility
(n = 21)

Records identified through PubMed and Embase database searching
(n = 163)

Additional records identified through, VIP, Wangfang, and CNKI database searching
(n = 21)

Records after duplicates removed
(n = 71)

## Identification

## Eligibility

## Screening
