# Supplementary material for: Four common vitamin D receptor polymorphisms and coronary artery disease susceptibility: A trial sequential analysis
Source: PLoS One. 2022 Oct 3;17(10):e0275368. doi: 10.1371/journal.pone.0275368 (PMC9529108; doi:10.1371/journal.pone.0275368)
Supplement: S2 Table — (DOCX) [file pone.0275368.s002.docx]

| Table S2. Methodological quality assessment for included studies. | | | | | | | |  |  |
| --- | --- | --- | --- | --- | --- | --- | --- | --- | --- |
| Criteria | | | | | | | | | Score |
| 1. Representativeness of cases | | | | | | | | |  |
|  | Coronary artery disease diagnosed according to acknowledged criteria. | | | | | | | | 2 |
|  | Mentioned the diagnosed criteria but not specifically described. | | | | | | | | 1 |
|  | Not Mentioned. | | | | | | | | 0 |
| 2. Source of controls | | | | | | | | |  |
|  | Population or community based. | | | | | | | | 3 |
|  | Hospital-based coronary artery disease free controls. | | | | | | | | 2 |
|  | Coronary artery disease free controls with related diseases. | | | | | | | | 1 |
| 3. Sample size | | | | | | | | |  |
|  | ≥500 | | | | | | | | 2 |
|  | <500 | | | | | | | | 1 |
| 4. Quality control of genotyping methods. | | | | | | | | |  |
|  | Repetition of partial/total tested samples with a different method. | | | | | | | | 2 |
|  | Repetition of partial/total tested samples with a same method. | | | | | | | | 1 |
|  | Not described. | | | | | | | | 0 |
| 5. Hardy-Weinberg Equilibrium (HWE) | | | | | | | | |  |
|  | Hardy-Weinberg Equilibrium in control populations. | | | | | | | | 1 |
|  | Hardy-Weinberg Disequilibrium in control populations. | | | | | | | | 0 |
|  |  |  |  |  |  |  |  |  |  |
